# Descemet membrane endothelial keratoplasty (DMEK) adoption, surgical barriers, and graft customization preference among corneal surgeons: A cross-sectional survey

**DOI:** 10.1371/journal.pone.0349580

**Published:** 2026-05-21

**Authors:** Abdullrahman Mohammed Alshehri, José Vargas, Mohammed Almutlak, Rafah Fairaq, Sami T. Hameed, Halla Alabdulhadi, Sara AlHilali, Mohammed M. Abusayf, Halah Bin Helayel

**Affiliations:** 1 Anterior Segment Division, King Khaled Eye Specialist Hospital, Riyadh, Saudi Arabia; 2 College of Medicine, King Saud bin Abdulaziz University for Health Sciences, Riyadh, Saudi Arabia; 3 Ophthalmology Division, King Abdullah Bin Abdulaziz University Hospital, Riyadh, Saudi Arabia; 4 Department of Ophthalmology, College of Medicine, King Saud University, Riyadh, Saudi Arabia; Singapore National Eye Centre, SINGAPORE

## Abstract

**Objective:**

To assess surgeon preferences, challenges, and graft-size customization practices in Descemet membrane endothelial keratoplasty (DMEK), and to explore their association with surgical confidence and adoption.

**Methods and analysis:**

A cross-sectional, 22-item online survey was distributed to corneal surgeons to evaluate surgical experience, preferences, graft-size customization practices, and challenges related to DMEK adoption. A total of 55 complete responses were analyzed. Categorical variables were summarized as frequencies and percentages. Multiple-response items were analyzed independently. Selected variables were grouped into clinically meaningful categories. Exploratory subgroup analyses were performed based on DMEK case volume, and associations were assessed using Fisher’s exact test.

**Results:**

A total of 55 participants completed the survey, of whom 47 (85.45%) had performed DMEK. Most participants were male (44, 80.0%) and aged 30–39 years (24, 43.63%). Customized graft sizing was used by 35 (74.47%) participants, and 36 (76.60%) reported that it influenced surgical decision-making or outcomes. Graft unfolding and preparation were the most challenging steps, with 27 (57.45%) and 24 (51.06%) participants reporting moderate to high difficulty, whereas patient selection was generally less difficult (28, 59.57% reporting no difficulty). Higher competence was reported for Descemetorhexis (29, 61.70%) and tissue selection (27, 57.45%), while lower competence was observed for graft unfolding (15, 31.91%). Hands-on and supervised learning methods were most valued, including wet-lab training (32, 68.09%) and one-on-one operating room training (34, 72.34%). Increasing experience was associated with improved performance but was not statistically significant.

**Conclusion:**

DMEK adoption is influenced by both technical complexity and training exposure. While surgeons demonstrate confidence in foundational steps, graft preparation and unfolding remain key challenges. Structured hands-on training and supervised experience appear critical to improving surgical confidence and may support broader adoption of DMEK.

## Introduction

Corneal endothelial dysfunction is a leading indication for endothelial keratoplasty, with Fuchs Endothelial Corneal Dystrophy (FECD) and pseudophakic bullous keratopathy (PBK) following intraocular surgery as the most common causes [1]. Among endothelial keratoplasty techniques, Descemet membrane endothelial keratoplasty (DMEK) has gained increasing popularity due to its ability to selectively replace the host Descemet membrane (DM) and endothelium with donor DM and endothelium, preserving the recipient’s corneal stroma [2].

The adoption of DMEK has increased steadily in recent years. According to the Eye Bank Association of America’s 2023 Eye Banking Statistical Report, 17,116 DMEK procedures were performed in the United States in 2023. For comparison, there were 15,248 DMEK procedures in 2022 and 13,215 in 2019 [3]. The increasing popularity of DMEK can be attributed to its superior visual outcomes, lower graft rejection rates, and better restoration of corneal anatomy, closely resembling that of a normal cornea [4–8]. However, despite these advantages, DMEK presents technical challenges that can lead to complications such as graft detachment and the need for rebubbling, with complication rates decreasing as surgeons gain more experience [9–11].

Despite these advantages, many surgeons continue to face barriers to DMEK adoption, including a steep learning curve, technical difficulty, and limited experience [12–14]. In a survey by Varadaraj et al., among 118 participants, 48 reported not performing DMEK, most commonly due to technical difficulty and a steep learning curve (63%), lack of experience (60%), low surgical volume (42%), and concerns about postoperative complications (40%) [13].

A key factor in optimizing DMEK outcomes and reducing complications is graft size selection, which plays a crucial role in both recipient compatibility and long-term graft survival [15]. Customizing graft size requires consideration of multiple parameters. White-to-white (WTW) corneal diameter is commonly used as a practical reference; however, in more complex cases, additional factors such as anterior chamber (AC) dimensions and posterior corneal characteristics may be relevant for more precise planning [16–18].

Unlike previous surveys that have primarily explored general barriers to DMEK adoption, this study focuses on graft-size customization and graft-planning preferences, providing insight into how these factors relate to surgeons’ confidence and adoption.

This study aims to characterize current surgical practices, training backgrounds, and perceived challenges in performing DMEK. It also seeks to assess the extent to which graft-size customization influences surgeons’ interest in adopting and performing DMEK.

## Materials and methods

This cross-sectional study was conducted between 29 June 2024 and 10 October 2024 after obtaining ethical approval from the Institutional Review Board of King Khaled Eye Specialist Hospital, Riyadh, Saudi Arabia (approval number: RP 24073-P). The study adhered to the tenets of the Declaration of Helsinki. Participation was voluntary, and informed consent was obtained electronically prior to survey initiation. All responses were collected anonymously using a secure Google Forms platform.

A total of 713 questionnaires were distributed through professional ophthalmology networks, corneal surgeon mailing lists, and direct invitations using a snowball sampling approach. Fifty-five complete responses were received, yielding a response rate of 7.7%. Surgeons with a wide range of experience, including those without prior DMEK training, were included to better capture barriers to adoption and variations in training pathways.

The questionnaire was adapted from a previously published survey by Varadaraj et al. and revised by the investigators to include items related to graft-size customization, training pathways, perceived difficulty, competence, and learning methods [13]. It was reviewed by members of the study team with expertise in corneal surgery to ensure clarity and relevance prior to distribution. The final instrument consisted of 22 items covering demographic and professional background, surgical experience and training, graft-planning practices, perceived barriers, and learning preferences. The survey design and reporting were aligned with CHERRIES guidelines for online surveys where applicable.

For several items (e.g., training location, tissue type, graft-planning parameters, and perceived barriers), respondents were allowed to select multiple options. To minimize recall bias, responses related to surgical experience and training timelines were collected using predefined categorical ranges rather than exact values. All items were set as required fields to minimize missing data; however, participants could discontinue the survey at any time. Duplicate responses were minimized by restricting submissions to one entry per device.

Difficulty was assessed using ordinal categories (not at all difficult, a little difficult, moderately difficult, and very difficult). Competence was assessed using a four-level scale: level 1 (not competent), level 2 (able to perform with moderate supervision), level 3 (able to perform with minimal supervision), and level 4 (able to perform independently). Participants who had not performed DMEK were excluded from procedure-specific analyses. The full questionnaire is provided in S1 Appendix.

### Statistical analysis

Data were analyzed using Stata (version 16; StataCorp, College Station, TX, USA). Categorical variables were summarized as frequencies and percentages.

For survey items allowing multiple responses, each option was coded independently, and percentages were calculated based on the number of respondents selecting each option; therefore, totals may exceed 100%.

Exploratory subgroup analyses were performed by stratifying participants according to DMEK case volume (≤10, 11–50, and >50 cases). Key outcomes were simplified into binary categories (e.g., moderate/high difficulty vs. low difficulty; independent vs. non-independent competence) to facilitate comparison across experience levels.

Associations between experience level and these outcomes were evaluated using Fisher’s exact test. A p-value <0.05 was considered statistically significant.

## Results

### Demographic characteristics

Demographic and professional characteristics of the participants are summarized in Table 1. A total of 55 participants were included in the study. The largest age group was 30–39 years (24, 43.63%), and the majority were male (44, 80.0%). Most participants had received cornea fellowship training (51, 92.7%) and prior exposure to DMEK training. (45, 81.8%).

**Table 1:** Baseline demographic characteristics, training background, and keratoplasty surgical experience of the study participants (n = 55).

Variables	Number (%)
**Age categories**	
(30–39) Years	24 (43.63%)
(40–49) Years	12 (21.81%)
(50–59) Years	10 (18.18%)
(> 60) Years	9 (16.36%)
**Gender**	
Male	44 (80%)
Female	11 (20.0%)
**Cornea fellowship trained**	51 (92.7%)
**DMEK Trained**	45 (81.8%)
Location of DMEK Training*	
During fellowship training	25 (38.92%)
Hospital-based Training	13 (20.0%)
Self-taught	3 (4.62%)
Special courses	24 (36.92%)
Place of practice*	
Academic/university setting	26 (32.50%)
Private practice	41 (51.25%)
Public hospital	13 (16.25%)
**Surgeon level**	
Surgeon in practice	54 (98.18%)
Trainee Fellow	1 (1.8%)
**Surgical experience**	
**Number of penetrating keratoplasty procedures**	
1–10	5 (9.09%)
11–50	11 (20.00%)
51–100	23 (41.82%)
101–500	7 (12.73%)
≥501	9 (16.36%)
**Number of DSAEK/DSEK procedures**	
1–10	11 (20.0%)
11–50	21 (38.18%)
51–100	14 (25.45%)
101–500	5 (9.09%)
≥501	4 (7.27%)
Number of DMEK procedures	
I did not perform DMEK	8 (14.55%)
1–10	21 (38.18%)
11–50	12 (21.82%)
51–100	7 (12.73%)
101–500	4 (7.27%)
≥501	3 (5.45%)

**Abbreviations:** DMEK = Descemet membrane endothelial keratoplasty; DSAEK = Descemet stripping automated endothelial keratoplasty; DSEK = Descemet stripping endothelial keratoplasty.

*Multiple responses allowed; percentages may exceed 100%.*

The majority of participants were practicing surgeons (54, 98.18%), working predominantly in private practice (41, 51.25%) and academic settings (26, 32.50%).

Participants demonstrated a broad range of keratoplasty experience. Most reported moderate experience in penetrating keratoplasty, with the largest proportion performing 51–100 procedures (23, 41.82%). A similar pattern was observed for DSAEK/DSEK, where most participants reported performing 11–50 procedures (21, 38.18%).

With respect to DMEK, the largest proportion of participants had limited experience, with 21 (38.18%) reporting 1–10 cases, followed by 12 (21.82%) reporting 11–50 cases.

**Fig 1:**
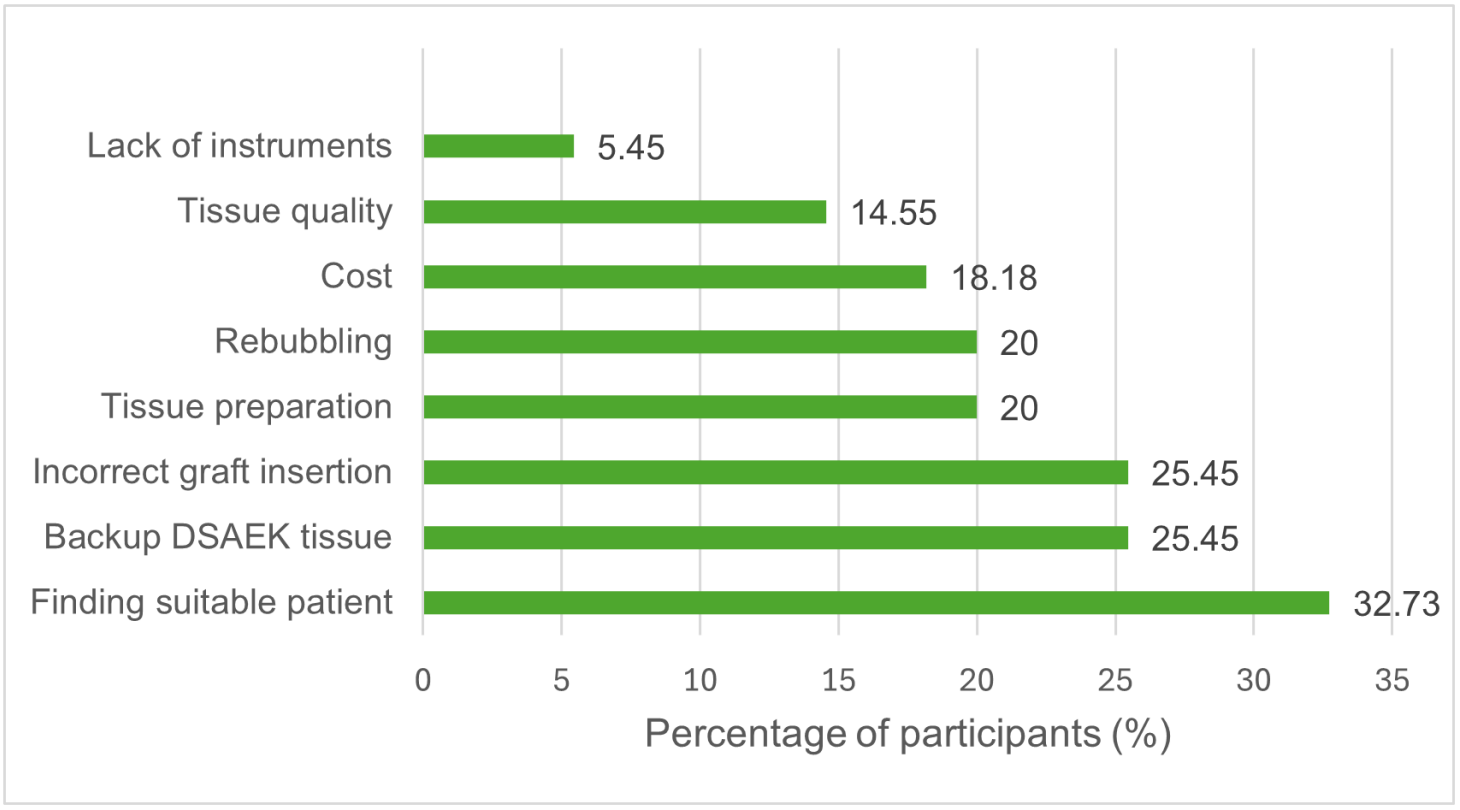
Barriers to DMEK adoption among participants (n = 55). Bars represent the percentage of participants identifying each barrier. Multiple responses were allowed.

Barriers to DMEK adoption are summarized in Fig 1. The most common barrier was difficulty identifying suitable patients for DMEK (18/55, 32.73%). This was followed by concerns regarding the need for backup DSAEK tissue and anxiety about incorrect graft insertion (14 participants each, 25.45%). Tissue preparation difficulty and concerns about rebubbling were each noted in 11 participants (20.00%). Cost-related concerns were identified in 10 participants (18.18%), while tissue quality concerns were less frequent (8 participants, 14.55%). A small proportion indicated lack of surgical instruments (3 participants, 5.45%).

Following assessment of perceived barriers, participants’ engagement with DMEK practice was evaluated. Of the 55 participants, 8 (14.55%) did not perform DMEK, whereas the remaining 47 (85.45%) reported performing DMEK with varying levels of surgical experience.

Subsequent analyses related to graft customization, surgical technique, training interval, perceived difficulty, and competence were restricted to participants who had performed DMEK.

### Training and surgical practice patterns

As shown in Table 1, the majority of participants had prior exposure to corneal and DMEK training. Participants reported multiple routes of exposure to DMEK training, most commonly during fellowship training (25, 38.92%) and specialized courses (24, 36.92%). Hospital-based training course was reported by 13 participants (20.0%), while 3 (4.62%) reported being self-taught.

**Fig 2:**
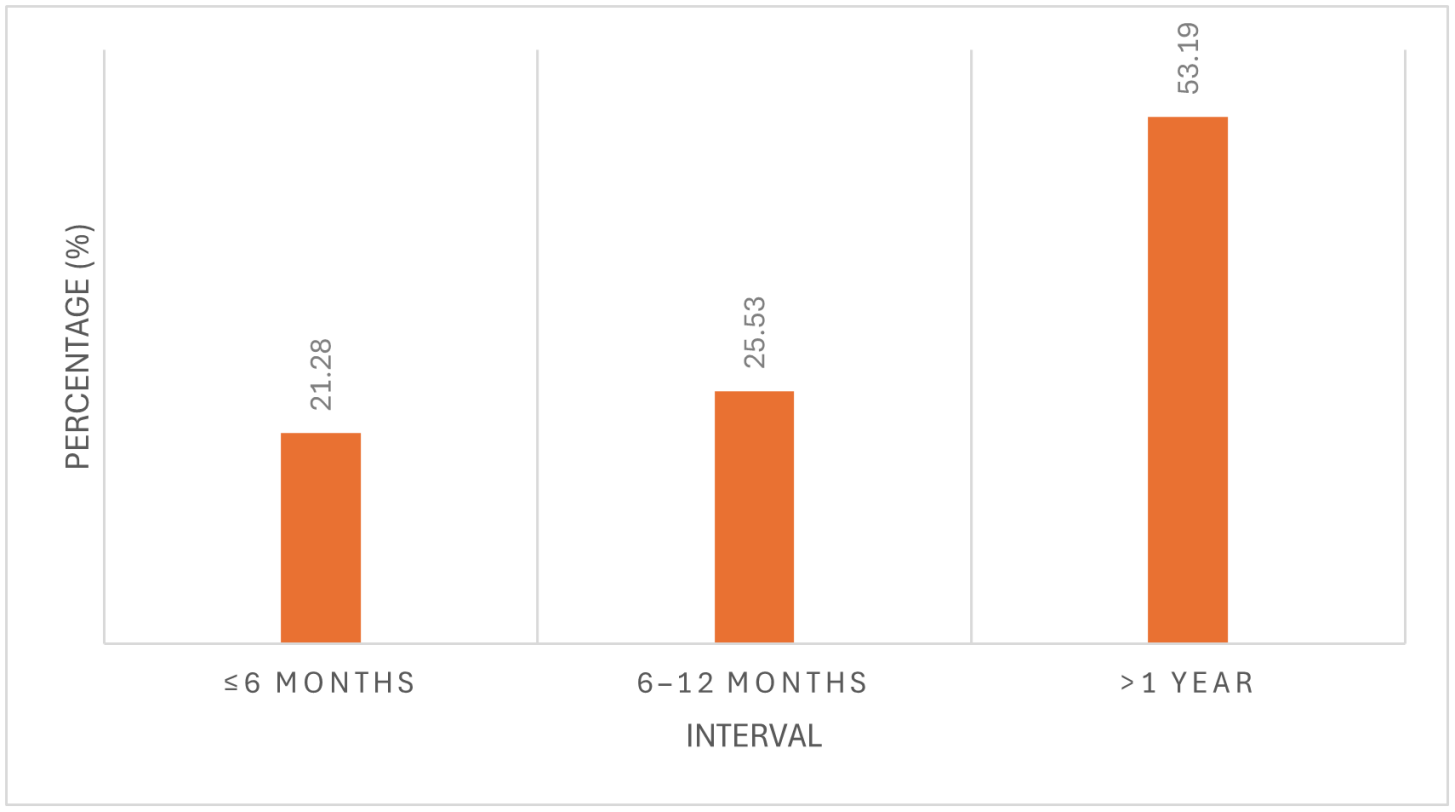
Interval between DMEK training and first surgical case among participants who had performed DMEK (n = 47). Bars represent the percentage of participants within each time interval.

Ten participants (21.28%) performed their first case within 6 months of training, while 12 (25.53%) did so between 6 and 12 months. The remaining 25 participants (53.19%) reported an interval exceeding one year (Fig 2).

Among participants who had performed DMEK (n = 47), 35 (74.47%) used customized graft sizing, whereas 12 (25.53%) used a uniform graft size. Consistently, most participants indicated that graft-size customization influenced their decision to perform DMEK or impacted surgical outcomes (36/47, 76.60%).

**Fig 3:**
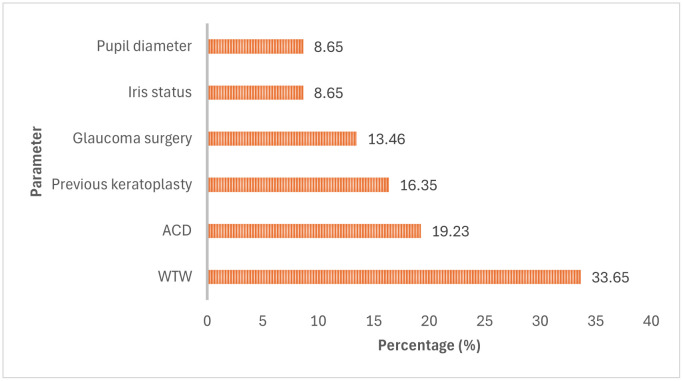
Parameters used for DMEK graft-size planning. Bars represent the percentage of participants selecting each parameter. Multiple responses were allowed.

Participants used multiple parameters to plan DMEK graft size (Fig 3). The most commonly used parameter was white-to-white (WTW) corneal diameter (35, 33.65%), followed by anterior chamber depth (20, 19.23%) and previous corneal transplantation (17, 16.35%). Participants also considered previous glaucoma surgery (14, 13.46%), iris tissue status (9, 8.65%), and pupil diameter (9, 8.65%).

Surgeon-cut tissue was the most commonly used graft type (26, 40.0%), followed by preloaded tissue (25, 38.46%) and precut tissue (14, 21.54%).The endothelium-out technique was the most commonly used method of DMEK insertion (81.13%), whereas the endothelium-in (pull-through) technique was used by 18.87% of participants.

### Perceived difficulty of DMEK surgical steps

**Fig 4:**
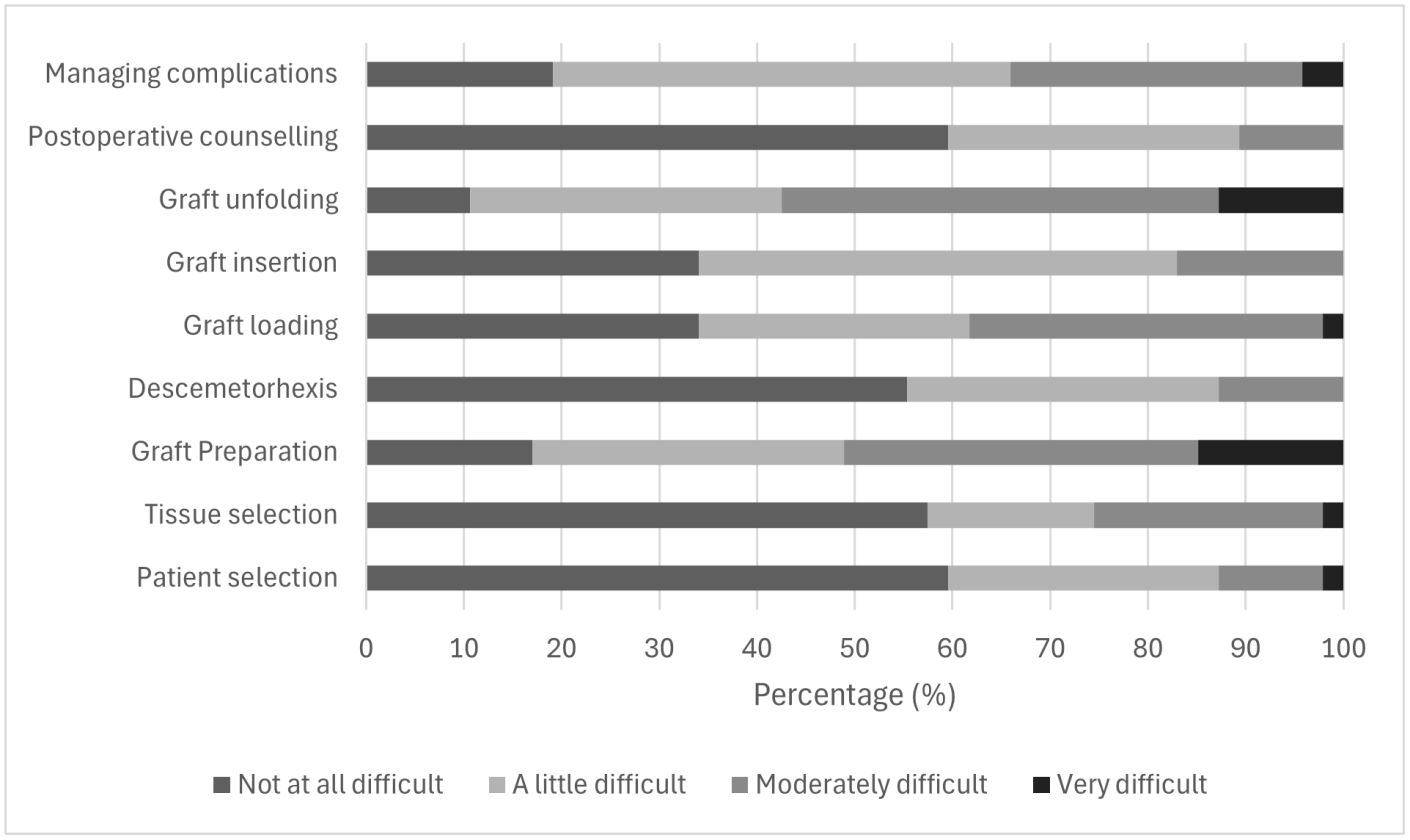
Perceived difficulty of different steps in DMEK surgery among participants who had performed DMEK (n = 47). Bars represent the distribution of responses across four difficulty levels: not at all difficult, a little difficult, moderately difficult, and very difficult.

Participants reported variable difficulty across different steps of DMEK surgery (Fig 4). Most initial and perioperative steps, including patient selection, tissue selection, Descemetorhexis, and postoperative counselling, were generally perceived as not difficult, with more than half of participants reporting no difficulty (e.g., patient selection: 28/47, 59.57%; tissue selection: 27/47, 57.45%).

In contrast, graft preparation and graft unfolding were the most technically challenging steps. Moderate to high difficulty was reported by 24 participants (51.06%) for graft preparation and 27 participants (57.45%) for graft unfolding.

Preparation for insertion and management of complications were also perceived as moderately challenging, with 18 (38.30%) and 16 (34.04%) participants reporting moderate to high difficulty, respectively. In contrast, graft insertion was generally considered less difficult, with most participants reporting either no or only a little difficulty (39/47, 82.98%).

### Self-reported competence across DMEK surgical steps

**Fig 5:**
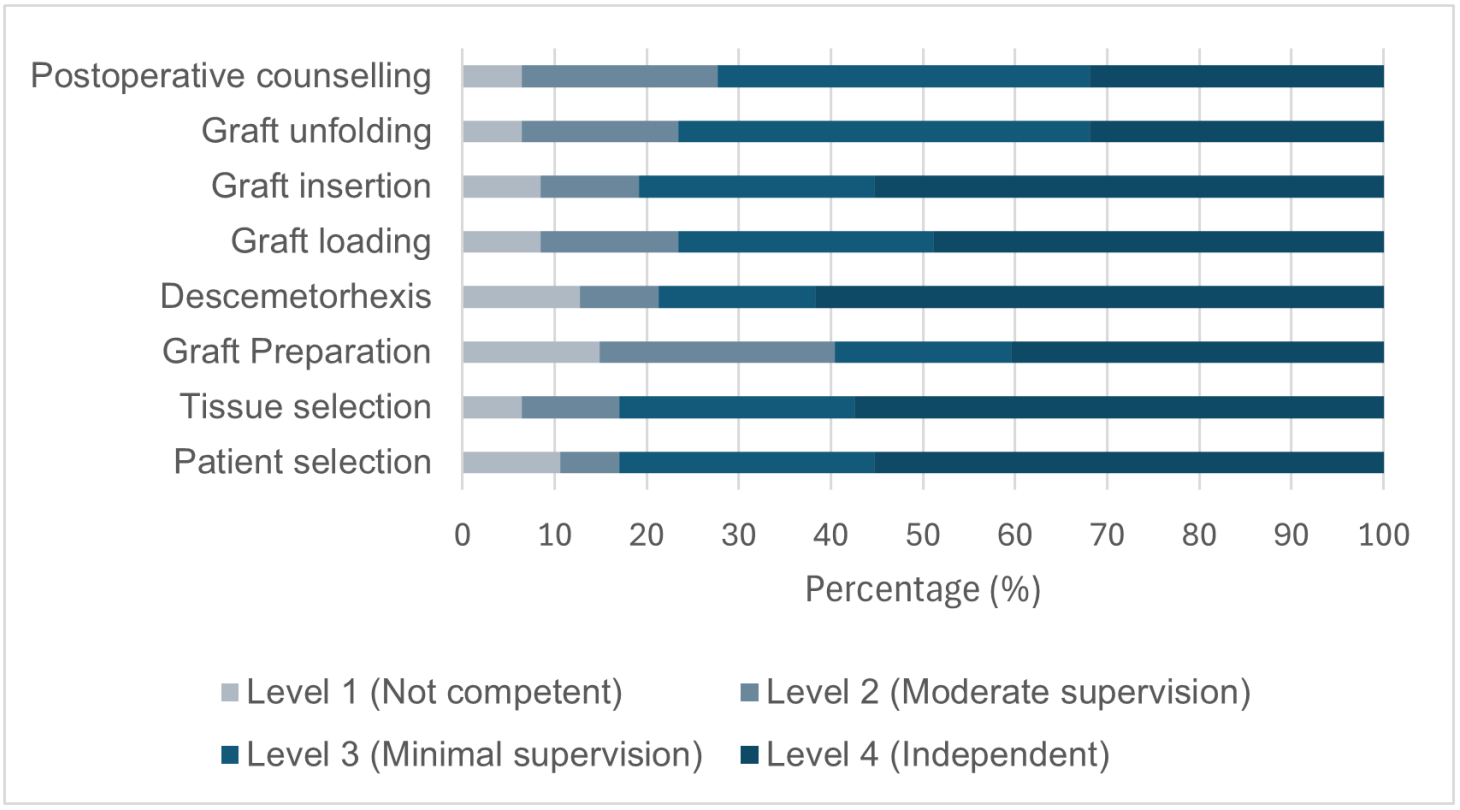
Self-reported competence across different steps of DMEK surgery among participants who had performed DMEK (n = 47). Bars represent the distribution of responses across four competence levels: level 1 (not competent), level 2 (able to perform with moderate supervision), level 3 (able to perform with minimal supervision), and level 4 (able to perform independently).

Participants reported variable levels of competence across different steps of DMEK surgery (Fig 5). Most participants achieved high competence (level 4) in patient selection (26/47, 55.32%), tissue selection (27/47, 57.45%), and Descemetorhexis (29/47, 61.70%).

In contrast, lower levels of competence were observed for graft preparation and unfolding. Only 19 participants (40.43%) reported level 4 competence in graft preparation, while unfolding remained the most challenging step, with the majority reporting level 3 competence (21/47, 44.68%) and only 15 (31.91%) achieving level 4 competence.

For graft insertion, 26 participants (55.32%) reported level 4 competence, whereas overall DMEK competence was most commonly reported at level 3 (19/47, 40.43%), with 15 participants (31.91%) achieving full independence (level 4).

The number of procedures required to achieve level 4 competence varied, with most participants reporting 11–20 cases (14/47, 29.79%) or 6–10 cases (13/47, 27.66%).

### Perceived value of learning methods for DMEK skill acquisition

**Fig 6:**
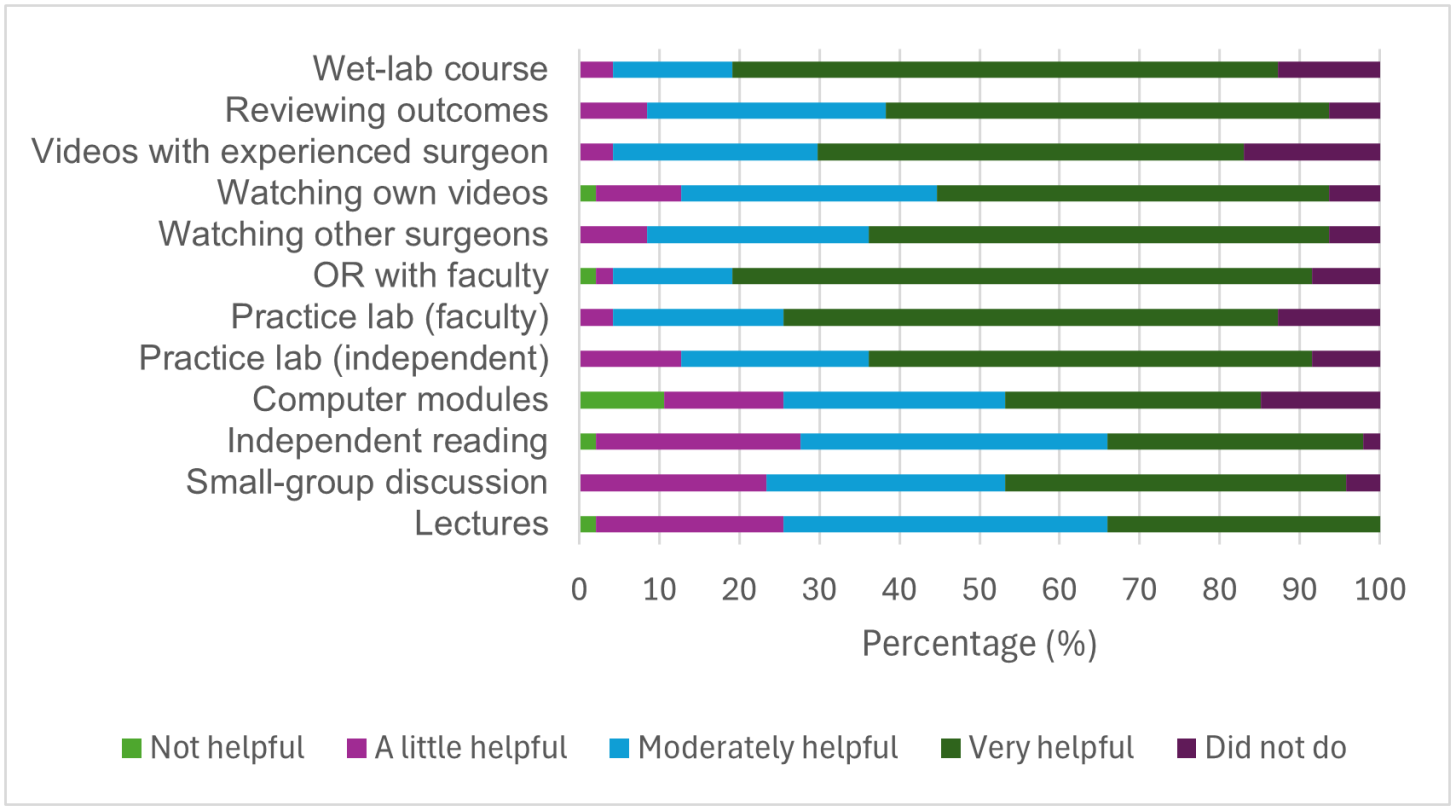
Perceived value of different learning methods for developing DMEK surgical skills among participants who had performed DMEK (n = 47). Bars represent the distribution of responses across levels of usefulness (not helpful, a little helpful, moderately helpful, very helpful, and did not do).

Participants reported varying value across different learning methods in developing DMEK surgical skills (Fig 6). Independent hands-on training methods were perceived as the most valuable. Independent time in a practice lab was rated as very helpful by 26 participants (55.32%), and one-on-one interaction with faculty in the practice lab was rated as very helpful by 29 (61.70%).

Video-based learning was also highly valued. Watching surgical videos with an experienced surgeon was rated as very helpful by 34 participants (72.34%), while reviewing personal surgical videos was rated as very helpful by 23 (48.94%).

Attending wet-lab training courses was considered highly beneficial, with 32 participants (68.09%) rating it as very helpful.

In contrast, traditional learning methods such as lectures and independent reading were more frequently rated as moderately helpful, with 19 (40.43%) and 18 (38.30%) participants, respectively, reporting moderate benefit.

When stratified by DMEK surgical experience, participants with higher case volumes reported lower perceived difficulty and higher levels of competence in graft unfolding. Among surgeons with ≤10 cases, 73.33% (11/15) reported moderate to high difficulty, compared to 54.55% (6/11) in those with 11–50 cases and 47.62% (10/21) in those with >50 cases.

Similarly, independent competence (level 4) increased with experience, from 26.67% (4/15) in surgeons with ≤10 cases to 27.27% (3/11) in those with 11–50 cases and 38.10% (8/21) in those with >50 cases.

Although these differences did not reach statistical significance (Fisher’s exact test, p = 0.354 for difficulty and p = 0.782 for competence), a consistent trend toward improved performance with increasing surgical experience was observed.

## Discussion

DMEK has become a favored approach for treating endothelial corneal diseases [19]. One of its key advantages is the significantly lower graft rejection rate, attributed to the minimal amount of transplanted donor tissue and reduced immunogenicity [8]. Additionally, by sparing the donor’s stromal tissue, DMEK preserves corneal curvature, minimizes induced refractive error, and allows faster visual rehabilitation, with many patients achieving good visual outcomes within days to weeks [20–21]. These advantages help explain the increasing adoption of DMEK over earlier endothelial keratoplasty techniques [22].

Despite these advantages, adoption of DMEK remains limited by a steep learning curve. The fragility of the Descemet membrane makes graft preparation, handling, and implantation technically demanding, often requiring repeated exposure to achieve consistent outcomes [9]. Limited exposure to DMEK during training and lack of structured training programs further contribute to slow adoption. In addition, logistical challenges, including access to donor tissue and management of postoperative complications, may influence surgeon uptake [20].

In our study, graft unfolding was identified as the most challenging step, followed by graft preparation, reflecting the delicate nature of the Descemet membrane and the need for precise intraocular manipulation to achieve correct positioning. These findings highlight the importance of structured training and repeated hands-on experience in mastering critical steps of DMEK. Participants consistently identified wet-lab training, hands-on practice, and supervised surgical exposure as the most valuable learning methods. This aligns with previous reports showing that structured training programs, including wet labs and simulation-based courses, can improve surgical proficiency and confidence [23].

Our results are consistent with previous surveys examining barriers to DMEK adoption. Varadaraj et al reported that technical difficulty, limited experience, and concerns about complications were major barriers, similar to those identified in our cohort [13]. Unlike prior studies, our analysis adds a focused evaluation of graft-size customization and its relationship to surgeon confidence and adoption.

A clear preference for preloaded tissues and customized graft sizes was observed. Preloaded tissues may simplify workflow by eliminating intraoperative graft preparation and reducing procedural variability [24].

Customized graft sizing was commonly guided by white-to-white (WTW) measurements, although additional parameters such as anterior chamber depth and prior surgical history were also considered. While WTW remains a practical reference, relying on it alone may be insufficient in complex cases, where anatomical variability can influence graft behavior and surgical difficulty [16–18,25,26].

Anterior chamber depth (ACD) and posterior corneal characteristics are important considerations in DMEK graft customization, as they may reduce graft unfolding difficulty, facilitate attachment, and lower rates of graft detachment and endothelial cell loss (ECL) [17,25–27]. Prior corneal transplantation and glaucoma surgery may also influence graft-size selection, reflecting the need to account for altered anterior segment anatomy. However, there remains no clear consensus on optimal graft sizing in complex cases, and current practices vary [16–18,25,28].

In our study, the endothelium-out technique was more commonly used, consistent with its relative ease of preparation and loading [29]. However, this approach may expose the endothelium to greater manipulation and potential trauma. In contrast, the endothelium-in technique may facilitate more controlled unfolding and reduce intraocular manipulation, although it requires greater technical expertise during graft preparation [30].

This study has several limitations. First, the sample size was relatively small, with a response rate of 7.7%. Although low, similar response rates are reported in ophthalmology surveys, likely reflecting time constraints and clinical workload [13,31–33]. Second, the use of convenience and snowball sampling introduces potential selection bias, as surgeons with greater interest or experience in DMEK may have been more likely to participate. Third, the inclusion of participants with varying levels of DMEK experience, including those without prior exposure, may affect generalizability; however, this approach was intentional to better capture barriers to adoption. Finally, the cross-sectional design and reliance on self-reported data limit causal inference. These findings should therefore be interpreted as exploratory.

In conclusion, this study highlights the central role of structured training and hands-on experience in developing competence in DMEK surgery. Surgeons reported high confidence in foundational steps such as patient selection and Descemetorhexis, whereas graft preparation and unfolding remain key technical challenges. Preferences for graft-size customization and preloaded tissue were common and appear to influence surgical decision-making. While these findings are descriptive, they suggest that targeted training strategies and workflow optimization may support broader adoption of DMEK. Future studies with larger, more representative cohorts and validated assessment tools are needed to better define factors influencing surgical confidence and long-term adoption.

## Supporting information

S1 Appendix DMEK Questionnaire. Survey instrument used to assess demographic characteristics, training background, surgical experience, graft-size customization practices, perceived difficulty, self-reported competence, and learning methods among corneal surgeons performing Descemet membrane endothelial keratoplasty (DMEK).
(DOCX)

S1 Dataset Cleaned study dataset used for analysis. The file contains the anonymized and cleaned dataset used for the statistical analysis in this study, including all variables collected from the survey responses.
(XLSX)

## Abbreviations

AC: anterior chamber
ACD: anterior chamber depth
DM: descemet membrane
DMEK: descemet membrane endothelial keratoplasty
DSAEK: descemet stripping automated endothelial keratoplasty
DSEK: descemet stripping endothelial keratoplasty
ECL: endothelial cell loss
FECD: fuchs endothelial corneal dystrophy
LKP: lamellar keratoplasty
OR: operating room
PBK: pseudophakic bullous keratopathy
PK: penetrating keratoplasty
WTW: white-to-white
