# Supplementary material for: Descemet membrane endothelial keratoplasty (DMEK) adoption, surgical barriers, and graft customization preference among corneal surgeons: A cross-sectional survey
Source: PLoS One. 2026 May 21;21(5):e0349580. doi: 10.1371/journal.pone.0349580 (PMC13193538; doi:10.1371/journal.pone.0349580)
Supplement: S1 Appendix — Survey instrument used to assess demographic characteristics, training background, surgical experience, graft-size customization practices, perceived difficulty, self-reported competence, and learning methods among corneal surgeons performing Descemet membrane endothelial keratoplasty (DMEK). (DOCX) [file pone.0349580.s001.docx]

# Questionnaire appendix

# I. Characteristics of corneal surgeons who participated in the survey

1. **1. Age** *

*Mark only one oval.*

(27- 29) Years

(30- 39) Years

(40 – 49) Years

(50 – 59) Years

(> 60) Years

1. **2. Gender** *

*Mark only one oval.*

Male

Female

1. **3. Cornea trained** *

*Mark only one oval.*

Yes

No

1. **4. DMEK trained** *

*Mark only one oval.*

Yes

No

1. **5. If yes Where? (you can choose more than one option)**

*Check all that apply.*

Special course

Hospital Preparedness Training fellowship trained

Other:

1. **6. Type of tissue used for DMEK (you can choose more than one option)** *

*Check all that apply.* preloaded pre cut only surgeon cut I have not done DMEK

Other:

1. **7. Type of size** *

*Mark only one oval.* one size for all DMEK customize tissue size

- 1. have not done DMEK

1. **8. What parameters do you use to plan graft size? (you can choose more than one** * **option)**

*Check all that apply.*

Glaucoma surgery

Iris tissue

Pupil diameter

Anterior chamber depth

WTW

Previous corneal transplant (PKP, LKP, DSAEK, DMEK)

- 1. have not done DMEK

1. **9. What Methods of DMEK do you use? (you can choose more than one option)**

*Check all that apply.*

Endothelium-out (injection e.g. glass tube or cartridge )

Endothelium-in (pull-through)

1. **10. Do you think size customization of graft have impacted your decision to perform** * **DMEK/improve success rate?**

*Mark only one oval.*

Yes

No

I have not done DMEK

1. **11. When did you take the last DMEK wet-lab training (** interval between DMEK wet *

lab or training and first case performed?) *Mark only one oval.*

6 months

Between 6 months and 1 year

Between 1 and 2 years

Between 2 and 3 years

Between 3 and 4 years Between 4 and 5 years

>5 years

I have not done DMEK

1. **12. Current level of practice** *

*Mark only one oval.*

Surgeon in practice

Fellow

Other:

1. **13. Place of practice (you can choose more than one option)** *

*Check all that apply.*

Academic/university setting

Private practice

Combination of academic and private

Public hospital

Other:

1. **14. Place of practice** *Mark only one oval.*

KKESH

Other:

1. **15. Country of practice** *
2. **16. Formal surgical training in DMEK** *

*Mark only one oval.*

Yes

No

I have not done DMEK

Other:

# II. Surgical case volume of participants

18. **Surgeries performed** *

*Mark only one oval per row.*

0

1

–

10

11

–

50

51–100

–

101

500

≥501

Penetrating

keratoplasty

DSAEK/DSEK

DMEK

# III. Barriers limiting surgeon adoption of DMEK (you can choose more than one options

**1. What has been a barrier to preforming DMEK?** *

*Check all that apply.*

I have not started DMEK yet

Finding the right patient

Cost associated with DMEK tissue

Anxiety about tissue preparation, if preparing yourself

Anxiety about tissue quality, if using an eye bank

Concern about needing backup DSAEK tissue

Anxiety about increased possibility of rebubbling with initial DMEK

Anxiety about inserting the tissue incorrectly and needing to regraft

Do not believe the challenges associated with DMEK are worth switching from DSAEK

Lack of some surgical instruments or a set

# IV. Self-perceived difficulty with learning different steps of DMEK surgery

# How difficult did you find it to learn the following steps of DMEK surgery? * *Mark only one oval per row.*

Not at

all

difficult

A little

difficult

Moderately

difficult

Very

difficult

Selecting the

right patient

Selecting the

right tissue source if pre-

prepared

Preparing the

DMEK graft

Performing

descemetorhexis

Preparing the

DMEK tissue

for insertion

Inserting the

DMEK tissue

Unfolding the

DMEK tissue

Counselling the

patient

postoperatively

Managing

complications

**V. Self-reported surgeon competence regarding different steps of DMEK surgery How competent do you currently feel about performing each of the following steps of** * **surgery?**

*Mark only one oval per row.*

Level 1:

Not

competent

Level 2:

Able to do

with

moderate

coaching

from a

more

experienced

surgeon

Level 3:

Able to do

with

minimal

input from a

more

experienced

surgeon

Level 4:

Able to do

without

input from a

more

experienced

surgeon

Selecting the

right patient

Selecting the

right tissue source if

prepared

Preparing the

DMEK graft

Performing

descemetorhexis

Preparing the

DMEK tissue

for insertion

Inserting the

DMEK tissue

Unfolding the

DMEK tissue

How competent

do you feel in

DMEK surgery overall?

**In order to reach level 4 competence for DMEK surgery overall, how many** * **procedures did you need?**

*Mark only one oval.*

0–5

6–10

11–20

21–30

31–50

50+

# VI. Utility of various educational resources in improving surgeons' skills

# Rate the value of the following learning methods in building your surgical skills * *Mark only one oval per row.*

Not

helpful

A little

helpful

Moderately

helpful

Very

helpful

Did not

do

Lecture

Small-group

case-based

discussion

Independent

reading

Interactive

computer based

modules

Independent

time in a

practice lab

One-on-one

Interaction

with faculty

in the practice

lab

One-on-one

interaction

with faculty in the OR

Watching

videos of

other surgeons by myself

Watching my

own surgical

videos by

myself

Watching videos

(my own or other) with an attending

Reviewing my

Own surgical outcomes

Attending

wet-lab training course
